# Propofol in ICU Settings: Understanding and Managing Anti-Arrhythmic, Pro-Arrhythmic Effects, and Propofol Infusion Syndrome

**DOI:** 10.7759/cureus.40456

**Published:** 2023-06-15

**Authors:** Jananthan Paramsothy, Sai Dheeraj Gutlapalli, Vijay Durga Pradeep Ganipineni, Isabelle Mulango, Ikpechukwu J Okorie, Divine Besong Arrey Agbor, Crystal Delp, Hanim Apple, Borislav Kheyson, Jay Nfonoyim, Nidal Isber, Mallikarjuna Yalamanchili

**Affiliations:** 1 Internal Medicine, Richmond University Medical Center Affiliated with Mount Sinai Health System and Icahn School of Medicine at Mount Sinai, Staten Island, USA; 2 Internal Medicine Clinical Research, California Institute of Behavioral Neurosciences & Psychology, Fairfield, USA; 3 Internal Medicine, Thomas Hospital Infirmary Health, Fairhope, USA; 4 General Medicine, Sri Ramaswamy Memorial (SRM) Medical College Hospital and Research Center, Chennai, IND; 5 General Medicine, Andhra Medical College/King George Hospital, Visakhapatnam, IND; 6 Pulmonary and Critical Care, Richmond University Medical Center Affiliated with Mount Sinai Health System and Icahn School of Medicine at Mount Sinai, Staten Island, USA; 7 Electrophysiology, Richmond University Medical Center Affiliated with Mount Sinai Health System and Icahn School of Medicine at Mount Sinai, Staten Island, USA; 8 Anesthesiology, Richmond University Medical Center Affiliated with Mount Sinai Health System and Icahn School of Medicine at Mount Sinai, Staten Island, USA

**Keywords:** anaesthesia management, life threatening arrhythmia, anti-arrhythmia, propofol based sedation, propofol infusion syndrome

## Abstract

Propofol has revolutionized anesthesia and intensive care medicine owing to its favorable pharmacokinetic characteristics, fast onset, and short duration of action. This drug has been shown to be remarkably effective in numerous clinical scenarios. In addition, propofol has maintained an overwhelmingly favorable safety profile; however, it has been associated with both antiarrhythmic and proarrhythmic effects. This review concisely summarizes the dual arrhythmic cardiovascular effects of propofol and a rare but serious complication, propofol infusion syndrome (PRIS). We also discuss the need for careful patient evaluation, compliance with recommended infusion rates, and vigilant monitoring.

## Introduction and background

Propofol (2,6-diisopropyl phenol) is a revolutionary anesthetic agent developed by Imperial Chemical Industries Limited (London, UK). Since its introduction and approval in Europe in 1986 and 1989 in the United States, it has transformed anesthesia and intensive care unit (ICU) practices to the greatest extent possible [1,2]. Over the centuries, volatile anesthetic agents, such as ethers, chloroform, and nitrous oxide, have been used as anesthetic agents. However, volatile anesthetic agents pose challenges in the induction and maintenance of anesthesia. During anesthesia induction, volatile anesthetics can cause airway irritation, coughing, breath-holding, and laryngospasm [3]. Furthermore, inhaled anesthetics pose a challenge during the maintenance of anesthesia and immobility during surgery because of their unpredictable pharmacokinetics in maintaining anesthetic depth [4].

Volatile anesthetics can cause malignant hyperthermia, nephrotoxicity, and hepatotoxicity, highlighting the need for vigilant patient monitoring [5,6]. Propofol has solved the need for short-acting intravenous anesthetics to overcome these challenges. Propofol is sufficient for the induction and maintenance of anesthesia [7]. Owing to its rapid onset of action, it has become the standard drug of choice for induction. Anesthesia can be maintained using intermittent boluses or continuous infusions [7]. It became the standard drug of choice to achieve total intravenous anesthesia (TIVA), which was popular in ambulatory surgery and outpatient settings in the early 1990s. Other advantages of propofol include rapid recovery even after long periods of anesthesia and conscious sedation [7,8].

In modern medicine, propofol is the gold standard for the induction and maintenance of anesthesia, procedural sedation, and ICU sedation [9]. Propofol has become an indispensable tool in modern medicine because of its versatility and favorable pharmacokinetic profile.

Methodology

For our review of the literature, we collected articles from PubMed, PubMed Central, and Google Scholar. Keywords used were “propofol”, “arrhythmias”, "propofol-induced arrhythmias”, and "propofol cardiovascular effects." We used the MeSH search strategy to gather the data. We selected all relevant articles from their inception to May 15, 2023. We retained 85 articles, book chapters, books, and other essential publications for review.

## Review

Propofol in the ICU: versatile sedation for diverse clinical needs and improved outcomes

Stress-induced responses in the ICU environment, such as hemodynamic and metabolic changes, irritation of the endotracheal tube, sleep deprivation, and pain, can cause delirium [10]. Therefore, providing adequate sedation and analgesia in ICU settings is vital to prevent the risk of anxiety and agitation [11]. Furthermore, it is essential to assess the depth and quality of sedation to meet the changing needs of patients in the ICU setting. Therefore, several sedation scales have been developed and used to monitor the depth and quality of sedation in the ICU (for example, the motor activity assessment scale (MAAS), Ramsay agitation sedation scale (RASS), and Riker sedation-agitation scale (SAS]) [12]. One of the primary uses of propofol in the ICU is to facilitate mechanical ventilation and prevent agitation using sedation scales [12]. Adequate propofol sedation was achieved with fewer side effects than midazolam in randomized controlled trials (including after cardiac surgery) [11].

Propofol in Comparison to Other Commonly Used Anesthetic Agents

When compared to midazolam, patients who received propofol sedation awakened more quickly because propofol was redistributed to peripheral tissues (muscles and fat) and cleared metabolically. After stopping the infusion, patients usually awaken within 10 to 15 minutes and recover from the sedation due to the shorter half-life of propofol which is approximately 30 to 60 minutes [11,13]. Propofol has reduced clearance and volume of distribution in elderly patients; therefore, a lower dose is needed [14]. However, the pharmacokinetic parameters of propofol anesthesia did not differ significantly between patients with chronic renal and liver failure and those with normal liver and kidney function [14,15]. Furthermore, a study showed that propofol combined with clonazepam effectively treated refractory epilepticus, thereby reducing long-term neurological damage [16]. Propofol showed similar efficacy and outcomes as dexmedetomidine in mechanically ventilated patients with sepsis. A large multicenter trial concluded that propofol when compared to dexmedetomidine did not show a difference in outcomes between the two drugs, including days alive without delirium or coma, ventilator-free days, without death at 90 days, or cognitive status at six months [17]. Additionally, a single-center open-label prospective study showed that propofol-remifentanil compared to a midazolam-fentanyl regimen reduced mechanical ventilation days and allowed earlier discharge from the ICU after major cardiac surgery [18]

Neuroprotective Effects of Propofol

Propofol has neuroprotective effects, especially when used in the ICU for patients with head injuries. Studies have shown that in patients with traumatic brain injury, propofol infusion can help maintain or reduce intracranial pressure (ICP) and can increase mean cerebral perfusion pressure (CPP) (defined as mean arterial pressure (MAP) minus ICP ) above 60 mmHg [19-21]. Additionally, studies have shown neuroprotective effects by decreasing cerebral blood flow velocity (CBFV) by up to 35% and cerebral oxygen extraction (COE) by up to 10% in patients undergoing cardiac bypass surgery with a moderate hypothermia protocol. Furthermore, propofol improved dynamic cerebral autoregulation while decreasing COE by creating excess cerebral blood flow compared to cerebral oxygen demand, providing net neuroprotective effects [22]. Studies have shown a marked reduction in ICP when propofol is administered to patients undergoing craniotomies, particularly to those with high ICP before surgery [23,24]. Propofol provides effective ICU sedation for adults with rapid and predictable recovery times. Due to its advantages, propofol is suitable for a wide range of clinical settings, including mechanical ventilation, treatment of refractory epilepticus, and prevention of delirium, offering reliable sedation options.

Exploring the dual arrhythmogenic effects of propofol on the heart

Propofol and its arrhythmic properties are double-edged swords owing to their proarrhythmic and antiarrhythmic properties. We compiled a series of case reports covering the wide-spectrum effects of propofol on cardiac conduction.

Anti-arrhythmic Properties

Miró et al. reported a case in which a patient came to the emergency department (ED) with atrial fibrillation with rapid ventricular response converted back to sinus rhythm after receiving propofol for sedation while awaiting electrical cardioversion [25]. A case report was published in the British Journal of Anesthesia of a 68-year-old man who came to the ED with chest pain and palpitations; the initial electrocardiogram (EKG) showed supraventricular tachycardia (SVT), which was resistant to carotid sinus massage and intravenous adenosine was eventually converted to sinus rhythm after propofol administration during anesthesia induction while preparing for cardioversion [26].

In the case of a 76-year-old man with an extensive cardiac history of multiple myocardial infarctions coupled with dilated ischemic cardiomyopathy who presented to the ED with multiple ICD discharges and polymorphic ventricular tachycardia (VT), the polymorphic VT was resistant to intravenous medications including amiodarone, lidocaine, and metoprolol. But, propofol induction was successful in converting the patient's polymorphic VT to sinus rhythm [27].

Propofol was observed to normalize the conduction abnormalities in Wolff-Parkinson's white (WPW) syndrome with the disappearance of delta waves and shortening of the PR interval. In the case of a 29-year-old woman with WPW syndrome, pre-operative EKG showed delta waves and short PR intervals. After propofol induction, the delta wave disappeared, and the PR interval also normalized. Normal EKG conduction persisted until anesthesia was discontinued but post-operatively, the delta wave returned which clearly implied the transient nature of the anti-arrhythmic effect [28]. Propofol has been shown to reduce the incidence of reperfusion arrhythmias. During myocardial infarction, reperfusion of the heart can lead to potentially lethal arrhythmias, but propofol alleviates these reperfusion arrhythmias [29]. In animal experiments on guinea pigs with simulated cardiac ischemia and reperfusion, propofol has been shown to prevent reperfusion arrhythmias. Furthermore, propofol prevented ischemia-induced shortening of the action potential duration (APD), reduced dispersion of the action potential, decreased conduction blocks, and reduced reperfusion-induced ventricular arrhythmias [30].

Wu et al. studied the direct effects of propofol on the cardiac conduction system. Their research has shown that propofol significantly prolongs atrioventricular node (AV node) conduction and suppresses cardiac ion channels such as ICa (calcium ion channels), INa (sodium ion channels), and Ito (transient outward current channel) in a dose-dependent manner [31]. They also mentioned the therapeutic antiarrhythmic properties of propofol and special considerations when using it as an anesthetic agent in cardiac patients [31].

Pro-arrhythmic Effects

It was observed in a small-scale study that during radiofrequency catheter ablation (RFA) of 60 children with paroxysmal SVT, administration of propofol significantly slowed the conduction of the AV nodes compared to isoflurane [32]. In another case, a 43-year-old woman suffering from Plummer's disease was given general anesthesia via fentanyl and propofol; this combination produced more sympathetic suppression leading to sinus arrest during the procedure [33]. Various case studies have reported the possibility of ventricular arrhythmias caused by propofol boluses. An interesting case report illustrated this phenomenon; a 28-year-old man with long QT syndrome experienced ventricular arrhythmia during wound debridement surgery for osteomyelitis; it was speculated that sympathetic stimulation from wound irrigation and propofol bolus may have contributed to its development [34].

Reports of chronic propofol abusers being admitted to the ICU after becoming unconscious with a butterfly needle in the arm have also been seen in the past. On admission, the EKGs usually revealed typical characteristics of the Brugada syndrome, including changes in the leads V1-V3, hypotension, and metabolic acidosis. In one such instance despite treatment, the patient's condition worsened; EKG showed a prolonged QT interval and an idioventricular rhythm, and the patient eventually died from complications related to these conditions [35]. Another case study reported that a 78-year-old woman while undergoing closed reduction and displacement of the distal tibia under propofol sedation, developed an episode of torsade de pointes/polymorphic VT followed by ventricular fibrillation (VF) [36].

Association With Bradycardia

One study revealed that propofol could affect human atrial muscarinic receptors in a dose-dependent manner, potentially explaining its bradycardia-inducing effects [37]. A study in guinea pig hearts showed that propofol causes bradycardia dose-dependently by suppressing AV node conduction. Its negative dromotropic effect was found to be predominantly mediated by M2-muscarinic receptors [38]. One of the most extensive phase IV clinical trials with 25.981 patients on the hemodynamic effects of propofol showed that 4.8% of the patients experienced bradycardia, 42% occurring within the first 10 minutes. Furthermore, the trial showed that bradycardia is more pronounced when propofol is co-administered with beta-blockers or opioids [39].

Propofol mechanism of action and adverse effect pathophysiology

**Figure 1:**
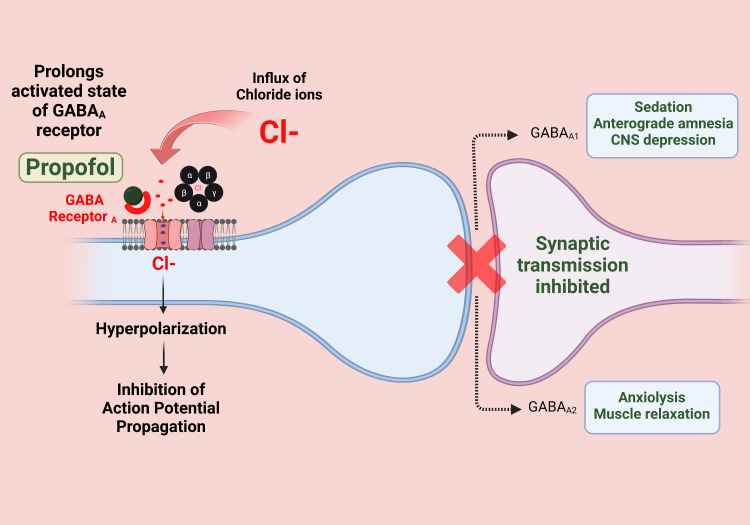
Propofol mechanism of action Image credits: Dr. Vijaya Durga Pradeep Ganipineni and Dr. Sai Dheeraj Gutlapalli

The action of propofol is mediated by an increase in the firing of GABAergic (gamma-aminobutyric acid) neurons in the ventrolateral preoptic area (VLPO). Binding to GABA receptors increases the duration and frequency of chloride channel opening. As a result, excitatory inputs are indirectly promoted to GABAergic neurons within the VLPO, increasing glutamate release and subsequent sedation [40]. The mechanism of action of propofol is shown in Figure 1. 

Effects on Cardiac Function and Electrophysiology

Propofol affects several critical aspects of cardiac electrophysiology. The cardioelectrophysiological system is based on ion flow through the calcium (Ca2 +), potassium (K +), and sodium (Na) channels. The K+ channels affected by propofol include ATP-sensitive channels and those responsible for delayed inward and transient outward rectification, which describe how ions flow through these channels. Owing to these interactions, propofol can influence APD [29].

Inotropic Effects

Propofol has been observed to have negative inotropic effects, it is in association with the drug's ability to decrease the heart muscle's contractile capacity. As a result, blood pressure and cardiac output decrease. Nevertheless, most in-vitro studies indicate that propofol exerts a minor inotropic effect at clinically relevant concentrations [41]. Looking at various case reports and studies regarding the inotropic effects gives us a mixed picture ranging from minor to significant inotropic effects especially considering animal studies.

In studies based on guinea pig myocytes, it was observed that propofol can result in a significant negative inotropic effect, due to blockade of the cardiac L-type calcium channel (LTCC). Subsequently, this action leads to a shortened APD and suppression of cardiac contractility [42]. However, this negative inotropic effect of propofol is detectable only at supra-therapeutic concentration. This effect can be explained by simultaneous compensatory mechanisms involving propofol blockade in the K + delay rectifier current (IKs) that counteract this effect at clinically relevant concentrations [43]. Another study involving guinea pig ventricular myocytes demonstrated that reduced APD and the amplitude of the inward calcium current were responsible for its negative inotropic effects [44].

Hanouz et al. demonstrated that propofol has different effects on right ventricular muscle strips in guinea pigs. These effects depend on the characteristics of the surrounding cells [30]. Under normal conditions, propofol shortens the APD, but this effect diminishes under ischemic conditions. It should also be noted that propofol is shown to reduce the variability of APD in areas transitioning between normal and ischemic zones under acute ischemic conditions. This could potentially reduce the incidence of spontaneous arrhythmias related to myocardial reperfusion injury [30]. Animal studies suggest that in normal myocardium, propofol does not impact cardiac contractility at therapeutic concentrations [45]. However, caution is advised when administering propofol to individuals with cardiac dysfunction. It was observed in various animal studies particularly canine studies that propofol-induced cardiac arrhythmias in hearts with pre-existing damage/dysfunction and it is hypothesised that a similar phenomenon can occur in patients with underlying cardiac dysfunction being more prone to arrhythmias with propofol administration [46].

Effects on Cardiac Ion Channels and Gap Junctions

There are two types of ATP-sensitive K+ channels (KATP), which are the mitochondrial KATP (mitoKATP) channels and sarcolemmal KATP (sarcKATP) channels. SarcKATP channels play a key role during phase 3 (rapid repolarization) and phase 4 (resting potential) of the cardiac action potential. MitoKATPs remain poorly understood and are not directly involved in the cardiac action potential because they do not reside directly within cell membranes. However, these channels exert cardioprotective effects during myocardial ischemia, which provides cardioprotective benefits. When ATP levels decrease, these channels open, leading to potassium efflux, which hyperpolarizes and reduces excitability within the cells, protecting them from injury. In studies on rat ventricular myocytes, propofol appears to have a minimal impact on these channels at clinically relevant concentrations. At supratherapeutic concentrations, propofol affects the activity of the KATP channels and mitochondrial oxidation [47].

The human heart possesses delayed rectifier K+ currents, which are essential for heart repolarization. These currents can be classified into three types: ultra-rapid (IKur), rapid (IKr), and slow (IKs) [48,49]. The IKr channel is responsible for rapid activation, inactivation, and prominent inward rectification. IKr is responsible for phase 3 of repolarization and is the target of Class III antiarrhythmic [29]. However, very few studies have investigated the effects of propofol on the IKr channels. A study in guinea pig ventricular myocytes revealed that propofol has no effect on IKr channels; instead, it predominantly affects the slow component of IKs channels [50]. Propofol inhibits delayed rectifier slow K+ currents (IKs), which are responsible for the final repolarization phase of the cardiac action potential (phase-3) in the atria and ventricles [43,49].

In phase 1 of the cardiac action potential, the transient outward rectifier channel current K + (Ito) contributes to rapid initial repolarization [48,49]. In rat ventricular myocytes, propofol at 25 and 50 mumol/L significantly reduced K+ (Ito) currents, potentially contributing to its therapeutic effects in certain cardiac arrhythmias [51].

According to Yang et al. (2015), propofol affects multiple potassium channels, including atrial K+(Ito) and IKur, and genes such as hKv1.5, hERG, and hKCNQ1/hKCNE1 are expressed in human embryonic kidney cells [48]. Propofol can inhibit KIto and IKur channels and their respective subunits, hKv1.5, and hERG, interfering with the initial and final phases of repolarization. Furthermore, inhibiting hKCNQ1/hKCNE1, which forms the slow delayed rectifier potassium channel (IKs), may affect the final repolarization phase of the cardiac action potential, possibly slightly prolonging atrial APD [48]. Inhibition of these atrial potassium channels by propofol and prolongation of the atrial action potential lead to anti-arrhythmic effects in the atrial myocardium [48].

Impact on Cardiac Sodium and Calcium Channels

Phase 0 of the cardiac action potential is initiated by an inward depolarizing current (INa) transmitted by the cardiac Na+ channels [49]. Propofol interacts with Na+ channels in a dose- and frequency-dependent manner, leading to a decrease in the inward Na + current and altered channel inactivation and recovery in ventricular myocytes. These changes contribute to the effects on the rate and rhythm (dromotropic and chronotropic effects) of the cardiac electrical conduction system and the prolongation of the conduction intervals at the AV node [31].

As mentioned above, propofol inhibits LTCC, which is essential for coupling cardiac cell excitation with cardiac contraction. LTCC channels are responsible for the plateau phase (phase 2) of the cardiac action potential, and there is a balance between the inward (calcium) and outward (potassium) currents. This creates a sustained depolarization, or "plateau” [49]. Furthermore, propofol has been shown to inhibit LTCC channels in guinea pigs, dogs, and rats' ventricular myocytes at clinically relevant concentrations [42,52-54].

Interactions With Connexin 43 and Implications for Ischemia-Induced Arrhythmogenesis

Gap junctions, small channels that connect cardiac cells, are composed of proteins called connexins. Connexin and connexin 43 (Cx43) facilitate electrical and metabolic communication between cardiac myocytes. This interaction facilitates the propagation and synchronization within the ventricle myocardium [29,55,56].

Hirata et al. (2000) examined the impact of anesthetics, specifically propofol and sevoflurane, on survival rates and the incidence of ventricular arrhythmias during acute myocardial ischemia, especially anesthetics impact Cx43, during MI [57]. Animal studies have shown that under reversible ischemia, increased extracellular resistance slows electrical conduction, while rapid uncoupling after 10-15 minutes can contribute to arrhythmias [58]. However, the study by Hirata et al. demonstrated that during 5-10 minutes after ischemia time, the most lethal arrhythmias occurred [57]. In their study, they noted propofol preconditioning as a possible means of mitigating potentially lethal arrhythmias after acute myocardial infarction [57]. Hirata et al. (2000) noted that propofol prevents lethal arrhythmia after myocardial infarction by protecting against phosphorylation of Cx43 and maintaining conduction of the gap junction [57].

Understanding Propofol’s Association With Bezold-Jarisch Reflex

The Bezold-Jarisch reflex comprising three symptoms (apnea, bradycardia, and hypotension) serves as an index of vagal nerve activity; however, its exact relationship to propofol-induced bradycardia remains elusive, with conflicting findings in human and rabbit studies [29,59-61]. Further research is necessary to clarify this relationship and understand the implications for patients receiving propofol.

Ikeno et al. demonstrated that propofol does not directly influence the cardiac conduction system in dogs through the autonomic pharmacological blockade, suggesting that its association with bradycardia is due to indirect mechanisms [62]. Propofol could protect against serious ventricular arrhythmias during acute coronary occlusion; however, this effect can be reversed using atropine Morey et al. showed that propofol antiarrhythmic effects are mediated by decreasing sympathetic tone and increasing parasympathetic dominance [29,63].

Table 1 details the effects of Propofol on the various cardiac ion channels. 

**Table 1:** Highlights the effects of propofol on cardiac ion channels and its effects Table credits: Dr. Jananthan Paramsothy

Cardiac ion channel	Function of ion channel	Overall effect on cardiac conduction
L-type Ca2+ (LTCC )	Responsible for phase 2 (plateau phase), critical for coupling cardiac cell excitation with contraction [49].	Inhibits the channels at clinically relevant concentrations, leading to a decrease in the inward Ca2+ current. This can result in a reduction in contractility, contributing to its negative inotropic effects [42, 52, 54].
K(ATP) (ATP-sensitive K+)	Play a role in phase 3 (rapid repolarization) and phase 4 (resting potential), and offer cardio-protection during myocardial ischemia [47].	Propofol does not have a significant effect on KATP channels at clinical concentration, thus no significant clinical effect is expected, At supra-therapeutic concentrations, propofol affects the activity of the K(ATP) channels and mitochondrial oxidation [47].
IKur (ultrarapid K+)	Early repolarization (phase 1) and late repolarization (phase 3) [49].	Prolongation of the duration of the atrial action potential leads to anti-arrhythmic properties in the atrium [48].
IKr (rapid K+)	Ventricular repolarization (phase 3) [49].	No significant changes [50].
IKs (slow K+)	Atrial and ventricular repolarization (phase 3) [49].	Propofol at certain concentrations significantly reduces K+ (Ito) currents, potentially contributing to its therapeutic effects in certain cardiac arrhythmias [48].
Ito (transient outward K+)	Contributes to rapid initial repolarization (Phase 1) [49].	Blockage potentially contributing to its therapeutic effects in cardiac arrhythmias [51].
Na channels	Initiate phase 0 with an inward depolarizing current (INa) [49].	Propofol interacts with these channels leading to a decrease in the inward Na+ current and altered channel inactivation and recovery. This affects rate and rhythm (dromotropic and chronotropic effects) of the cardiac electrical conduction system [31].
Cardiac gap junctions, composed of the protein Cx43	It enables cardiac myocytes to communicate electrically and metabolically with each other, thus aiding in impulse propagation and synchronization within the ventricular myocardium [49].	Prevention of lethal arrhythmias after myocardial infarction, by protecting against phosphorylation of Cx43 and maintaining conduction of the gap junction [51].

Propofol infusion syndrome (PRIS) and its clinical implications

Propofol can cause arrhythmias in lethal conditions such as PRIS, which are rare but potentially fatal complications if given for an extended period at high doses. PRIS may present as metabolic acidosis, rhabdomyolysis, acute kidney injury (AKI), fatty liver, hyperlipidemia, acute refractory bradycardia, and asystole [64]. Additionally, it can also cause lactate acidosis and myonecrosis by directly inhibiting the mitochondrial respiratory chain or altering mitochondrial fatty acid metabolism [65,66]. Several risk factors, such as young age, critical central nervous system or respiratory tract disorders, exogenous catecholamines and glucocorticoids, inadequate carbohydrate intake, and subclinical mitochondrial disease are associated with PRIS [66].

Association With Brugada Syndrome

Brugada syndrome is an autosomal dominant condition due to mutations in cardiac Na+ channels: a mutation of SCN5A results in accelerated inactivation of Na+ channels [67]. As a result of the rapid inactivation of Na+ channels, a voltage gradient is generated in the right ventricular muscle layers. This voltage gradient can trigger life-threatening VT or VF [68]. Brugada syndrome can be diagnosed by looking for specific ECG patterns (Type 1, 2, or 3) in one or more right precordial leads (V1-V3) on an EKG [69]. Specifically, the Brugada syndrome type 1 EKG pattern shows a coved ST-segment elevation >2 mm followed by a negative T wave; Type 2 and 3 EKG patterns have more variable morphology but still show saddleback ST-segment elevations [68]. However, these EKG patterns may not always be visible. They can be unmasked with sodium channel blockers such as ajmaline or flecainide [70].

As we discussed above, propofol inhibits cardiac Na+ channels. There have been multiple cases where propofol causes Brugada-like changes in the EKG [71]. During the early stages of PRIS, similar to Brugada syndrome, the right precordial leads (V1 to V3) show a block of the right bundle branch block with an elevation of the coved ST type [66]. Therefore, patients with Brugada syndrome should not receive propofol, as it can lead to life-threatening arrhythmias and sudden cardiac death. One of the striking characteristics of PRIS is myocardial failure, especially in children in the ICU receiving propofol infusion [72,73]. Jorens et al. reported the case of a 12-year-old boy who died due to PRIS; postmortem pathology reports showed cardiac myocytolysis and unprecedented widespread accumulation of myocardial fat accumulation, which explains myocardial failure caused by impaired free fatty acid utilization in cardiac muscle tissues [74]. Again, propofol is known to cause fatty acid oxidation disrupted and mitochondrial respiratory chain failure, leading to increased free fatty acid in the circulation [75]. Furthermore, studies have shown that free fatty acids are known to cause cardiac arrhythmias [76].

Preventing and Managing Propofol Infusion Syndrome: Strategies for Critical Care

To avoid PRIS, it is necessary to carefully evaluate the risks and potential benefits of propofol for sedation in critically ill patients, considering accessibility, economic feasibility, and potential sedative options [77]. PRIS requires early recognition and management based on clinical characteristics, followed by immediate cessation of propofol infusion and potential dextrose infusion to address possible mitochondrial involvement [77]. PRIS has been associated with high doses and prolonged infusion time [78,79]. Therefore, the current literature recommends against infusion rates exceeding 5 mg/kg/hr for more than 48 hours and recommends combining propofol with other sedatives, such as opioids [79,80]. Close monitoring of the pH, lactate, and creatine kinase (CK) levels during prolonged infusions, especially when high doses of sedation are warranted is essential to avoid PRIS, especially during prolonged infusion durations [80]. In managing PRIS, immediate therapeutic intervention should focus on mitigating life-threatening manifestations such as changes in the Brugada pattern of the EKG and other life-threatening arrhythmias, hyperkalemia, hypotension, and fever. Although acidosis may not directly cause death, it has the potential to induce cardiac arrhythmias and alter catecholamine efficacy [77]. Maximizing minute ventilation may offer patients with metabolic acidosis a means of compensating for their condition [81].

When standard treatments for hyperkalemia, acidosis, or fever do not respond satisfactorily, hemofiltration must be considered, and propofol administration should be discontinued in favor of alternative hypnotic agents such as dexmedetomidine or midazolam [82]. In cases of acute kidney injury due to PRIS, continuous hemofiltration can provide a significant advantage by eliminating the water-soluble metabolites of propofol, even though it does not eliminate its lipophilic parent compound [83]. Extracorporeal membrane oxygenation (ECMO) can be used as a last resort, and it has shown promising results when vascular compartment filling and vasopressors or inotropes are insufficient [73,84].

The literature suggests alternative treatments for PRIS. For example, a case study found that L-carnitine could help manage the condition by promoting fatty acid metabolism [85]. In this study, a 59-year-old ICU patient with PRIS due to extended low-dose propofol infusion quickly recovered after discontinuing propofol, receiving L-carnitine, and aggressive supportive care. The patient was eventually transferred from the hospital to rehabilitation. Within two days of starting L-carnitine treatment, the patient CK levels decreased, and the patient's condition stabilized [85].

Limitations

The important drawbacks of our literature review are that many of our findings are based on isolated and novel case reports with minimal corroborative evidence stemming from clinical trials; we focused on covering the wider spectrum of cardiac adverse effects that may stem from propofol use in the ICU and therefore discussed the rarest of rare topics which may or may not be common in the general day to day clinical ICU setting, Therefore we reiterate that many of these results are based on isolated case reports, case studies, and animal studies. These case studies included different patient populations and varying clinical scenarios. The possibility of unknown confounding factors and the lack of controlled studies limit our ability to draw definitive conclusions. At the same time, short-term observations and scarce data on alternative treatments such as L-carnitine can make understanding long-term implications and the efficacy of potential interventions more difficult. Future research should address these challenges to gain a comprehensive view of the impact and its management strategies in ICU settings.

## Conclusions

Propofol has profoundly affected the fields of anesthesia and intensive care medicine since its discovery in the late 20th century. Its favorable pharmacokinetic properties, rapid onset, and short-acting nature have made it a good agent for the induction and maintenance of anesthesia, procedural sedation, and sedation in the ICU. Its effectiveness in ICU settings is remarkable, as it offers rapid and predictable recovery times, reduced side effects, and versatility in various clinical scenarios. Furthermore, its neuroprotective effects make it invaluable for the management of patients with traumatic brain injuries and those undergoing craniotomies. Finally, propofol contributes to improved patient outcomes as a keystone for ICU sedation. 

Propofol has dual antiarrhythmic and proarrhythmic properties. Its antiarrhythmic properties are observed to be protective against reperfusion arrhythmias in some cases, but it has also been linked to ventricular arrhythmias, bradycardia, and prolonged APD. Propofol can cause rare complications such as PRIS when administered at high doses for extended periods. To avoid and manage PRIS, careful evaluation of the patient, adherence to recommended infusion rates, and close monitoring of patients taking propofol is essential. Early recognition of PRIS, immediate discontinuation of propofol, and interventions such as hemofiltration or ECMO can help mitigate life-threatening manifestations and improve outcomes in critically ill patients. Propofol has significantly transformed the landscape of anesthesia and intensive care practices, and it continues to be an indispensable tool for delivering safe and efficient anesthesia and sedation. Despite possible cardiovascular effects and rare complications, the safety profile of propofol in clinical settings remains generally favorable. To ensure optimal patient care, clinicians must be vigilant in identifying and managing the potential risks of arrhythmia and PRIS.

## Appendices

Important genes and subunits

hERG: Human Ether-a-go-go Related Gene (hERG) encodes for an alpha subunit of the rapidly activating delayed rectifier potassium channel (IKr). This channel plays an essential role in cardiac action potential repolarization phase and contributes to maintaining normal cardiac rhythm

hKCNQ1: Gene encoding alpha subunit of slow delayed rectifier potassium channel plays an integral part in cardiac action potential repolarization and has been linked with numerous cardiac conditions, such as long QT syndrome, Jervell-Nielsen Syndrome and familial atrial fibrillation

hKCNE1: Gene that encodes a transmembrane protein which acts as a regulatory subunit of the slow delayed rectifier potassium channel, playing an essential role in modulating channel function and associated with Jervell and Lange-Nielsen forms of long-QT syndrome as well as Romano-Ward forms of long-QT syndrome

SCN5A : Gene that encodes alpha subunit of tetrodotoxin-resistant voltage-gated sodium channel is essential in initiating action potential upstroke in cardiac muscle, as well as associated with various cardiac diseases

hKv1.5: Shaker-related genes encode alpha subunits of voltage-gated potassium channels that play an essential role in heart regulation by controlling resting membrane potential and action potential repolarization, with defects possibly leading to familial atrial fibrillation type

Cardiac channel functions

L-type Calcium Channel (LTCC): A channel that facilitates calcium ion entry into cardiac cells during the action potential plateau phase, playing a crucial role in muscle contraction and action potential prolongation

Cardiac sodium channel (INa): Essential for rapid depolarization in cardiac cells, initiating heart muscle contraction, and playing a role in various cardiac conditions while serving as a target for class 1 antiarrhythmic drugs

IKr (Rapid Delayed Rectifier Potassium Current): Essential for repolarization in cardiac cells, it assists in returning the membrane potential to its resting state

IKs (Slow Delayed Rectifier Potassium Current): Contributes to the repolarization phase in cardiac cells and plays a significant role in regulating the QT interval

IKur (Ultra-Rapid Delayed Rectifier Potassium Current): Participates in the repolarization phase of the action potential, specifically in atrial cells, and contributes to the atrial refractory period

Ito (Transient Outward Potassium Current): Involved in the early repolarization phase of the action potential in cardiac cells, helping to shape the action potential waveform

IK(ATP) (ATP-sensitive Potassium Channels): Regulates the resting membrane potential of cardiac cells in response to alterations in cellular ATP levels, which can be affected by metabolic stress or ischemia.
